# Prior to Conception: The Role of an Acupuncture Protocol in Improving Women's Reproductive Functioning Assessed by a Pilot Pragmatic Randomised Controlled Trial

**DOI:** 10.1155/2016/3587569

**Published:** 2016-05-03

**Authors:** Suzanne Cochrane, Caroline A. Smith, Alphia Possamai-Inesedy, Alan Bensoussan

**Affiliations:** ^1^School of Science & Health, Western Sydney University, Locked Bag 1797, Penrith, NSW 2751, Australia; ^2^National Institute of Complementary Medicine, Western Sydney University, Locked Bag 1797, Penrith, NSW 2751, Australia; ^3^School of Social Science & Psychology, Western Sydney University, Locked Bag 1797, Penrith, NSW 2751, Australia

## Abstract

The global average of couples with fertility problems is 9%. Assisted reproductive technologies are often inaccessible. Evidence points to acupuncture offering an opportunity to promote natural fertility. This study asked whether providing a multiphasic fertility acupuncture protocol to women with sub/infertility would increase their awareness of fertility and achieve normalisation of their menstrual cycle compared with a lifestyle control. In a pragmatic randomised controlled trial sub/infertile women were offered an intervention of acupuncture and lifestyle modification or lifestyle modification only. There was a statistically significant increase in fertility awareness in the acupuncture group (86.4%, 19) compared to 40% (*n* = 8) of the lifestyle only participants (Relative Risk (RR) 2.38, 95% confidence interval (CI) of 1.25, 4.50), with an adjusted *p* value of 0.011. Changes in menstrual regularity were not statistically significant. There was no statistical difference in the pregnancy rate with seven women (adjusted *p* = 0.992) achieving pregnancy during the course of the study intervention. Those receiving the acupuncture conceived within an average of 5.5 weeks compared to 10.67 weeks for the lifestyle only group (*p* = 0.422). The acupuncture protocol tested influenced women who received it compared to women who used lifestyle modification alone: their fertility awareness and wellbeing increased, and those who conceived did so in half the time.

## 1. Introduction

Fertility problems have become a major presenting condition in gynaecological clinics. Estimates of the number of couples encountering fertility problems vary from one in six to one in ten, with 9% currently cited as the probable global average [1]. As the biomedical response to infertility IVF remains “absent, inaccessible, or unaffordable for the majority of the world's infertile couples” [1, page 2], populations may utilise their traditional medical health systems. Chinese medicine has been used to treat female fertility problems using a range of methods throughout its history. In Western settings acupuncture is used as a primary intervention for fertility problems [2, 3]. Acupuncture is increasingly used as an adjunct to assisted reproductive technologies [4] and more widely in the complementary health care system.

Outside the context of assisted reproductive technologies (ART) clinics, there has been little research that supports the role of acupuncture in promoting women's reproductive health. Research of Chinese medicine's supportive contribution to fertility largely consists of case reports. In a survey of Australian and New Zealand acupuncturists [5] it was found that general fertility health treatments were the most common treatments administered to women. This emphasis in clinical practice has also been reported in the UK [6].

Clinical case reports support the value of acupuncture in the lead up to conception, although no clinical trial has been reported to date that either supports or contradicts this case-based evidence [7–10]. A prospective consecutive case series study of a course of 3 months of acupuncture for amenorrheic women with premature ovarian failure has found, compared to baseline, serum follicle stimulating hormone (FSH) and luteinising hormone (LH) were decreased (*Z* = 4.68, *p* = 0.001) and estradiol (E2) was increased (*Z* = 4.48, *p* = 0.001). Within the 3-month intervention approximately 20 percent of trial participants resumed their menstruation [11]. Chinese medicine texts and case history books, for example, frequently cite the use of acupuncture to induce ovulation. Early research reported that electroacupuncture induced ovulation in six out of 13 anovulatory cycles as well as higher hand skin temperature and lower blood radioimmunoreactive [beta]-endorphin concentrations in acupuncture induced ovulation cycles [12]. Gerhard and Postneek [13] found that infertile women with hormonal disturbances and anovulation treated with auricular acupuncture had similar pregnancy rates when compared with those treated with hormones.

The absence of previous research on applying acupuncture in the period of periconception for women who are having difficulty conceiving is identified as a research gap. This study sought to explore the potential contribution of an acupuncture protocol to enhancing female fertility.

## 2. Materials and Methods

The pilot study objective was to provide preliminary data to explore whether women with subfertility undergoing a course of acupuncture and lifestyle modification compared with an active control of lifestyle modification alone would demonstrate improved reproductive outcomes, improved menstrual cycles, and increased fertility awareness.

The study hypothesised that providing acupuncture to women with sub/infertility would increase their awareness of their fertility and achieve normalisation of their menstrual cycle compared with lifestyle as a control.

Secondary hypotheses were that acupuncture compared with lifestyle control would demonstrate reduced time from study entry to conception; increased clinical pregnancy rates; improved quality of life changes; and increased lifestyle change.

### 2.1. Participants and Recruitment

Participants were recruited from the community in Sydney, Australia between October 2009 and December 2011. Participants were initially recruited from existing contact lists within the research centre. Further recruitment sources were via media and social network advertising, such as local paper and Facebook and other internet sites. Also posters and pamphlets were distributed, within the university campuses and to other likely referral sources such as medical centres, chemists, and community centres. Women who made early contact also suggested further avenues for recruitment, their main suggestion being accessing the forums on fertility-specific websites. Several web forums agreed to include a statement about the trial and a request for women to contact the researcher for further information. A letter requesting referrals to the study was forwarded to women's health centres, medical centres, TCM clinics, and fertility centres in the target area of Western Sydney.

Ethics approval for this study was obtained from the Human Research Ethics Committee of University of Western Sydney: H7588. The trial was registered with the Australian and New Zealand Clinical Trial Registry as ACTRN12610000631000.

### 2.2. Inclusion/Exclusion Criteria

The inclusion criteria were women of reproductive age (18–44 years old) who had been actively trying to conceive (unprotected sex) without success (including miscarriage) for at least 12 months; who had a gynaecological diagnosis of the causes of their infertility; who are not planning to use acupuncture during the trial intervention; and who are able to attend at least 7 of 9 treatment sessions.

Gynaecological assessments and diagnoses were provided by participants based on their previous biomedical examinations prior to the study and were not reassessed at screening. Women were excluded if they met the criteria of having nonpatent fallopian tubes; absence of uterus; primary anovulation; or partner sperm defect (and not enrolled in IVF to use intracytoplasmic sperm injection (ICSI) to counter defect).

The randomisation sequence was computer generated by a researcher independent of the study based at the University of Western Sydney. The random allocation was sealed in opaque numbered envelopes held in sequential order and accessed by the acupuncturist in sequential order. Random allocation of participants was to either an acupuncture plus lifestyle intervention or lifestyle modification alone. As this was a pragmatic design there was no blinding of patient or acupuncturist. Data entry and analysis were undertaken blind to group allocation.

### 2.3. Intervention and Follow-Up

The intervention was administered over three months. The control of lifestyle support comprising diet and exercise is the most common first-stage intervention offered in general preconceptual care and is an appropriate base from which to assess fertility changes from an acupuncture intervention [15, 16]. Although lifestyle advice is a standard component of Chinese medicine care [17] a specifically targeted lifestyle intervention, focused on diet and exercise, provides a viable active comparator to an acupuncture intervention.

The diet was based on the CSIRO Total Wellbeing Diet [18] and is supported by research evidence that the diet optimises fertility treatment outcomes [15, 19]. The CSIRO Total Wellbeing Diet has been tested both in research and in the marketplace. It offers an explanation of a recommended diet and exercise regimen, detailed recipes, and a long-term maintenance plan. As this research project lacked the resources for individualised assessment and program design the CSIRO diet was adopted as it is considered nutritionally balanced. In its presentation the diet is accessible and sufficiently applicable to a range of women and has a health, rather than just a weight loss, focus. All participants were also asked to develop an exercise program within the guidelines offered by Noakes and Clifton to incorporate within their daily schedule. Those participants with already heavily weighted active exercise regime were asked to include softer exercise routines like meditation, yoga, Tai Chi, or walking and to reduce the amount of heavy exercise during their menses and ovulation. Those with little active exercise were encouraged to increase this, especially outside the times of menstruation and ovulation. Those who smoked cigarettes and/or drank alcohol or caffeine regularly were encouraged to develop a plan to reduce or remove them from their daily lives. Each participant was contacted by phone or email at least fortnightly or an attempt was made to have such contact by the study investigator to assess compliance with the lifestyle intervention.

In addition to the same lifestyle advice all participants randomised the acupuncture intervention received a Chinese medicine assessment. This was undertaken by the lead researcher who allocated each participant a TCM diagnosis and treatment strategy. The allocated diagnoses were forwarded to the treating acupuncturist. Acupuncture was administered using a manualised treatment protocol [20], predominantly within a TCM paradigm. The collective development of the protocol began from the foundation work of Lyttleton from Table 4.11 in the 2004 edition of her book ([14]: 158-9). Treatment was administered over three months with weekly acupuncture; acupuncture treatment was tailored and based on their TCM diagnosis, the phase of their menstrual cycle, an assessment of their spirit or emotional state, and biomedical condition. Their TCM diagnosis and phase of their menstrual cycle were given greatest priority followed by their presenting emotional state, their biomedical diagnosis, and specific presenting signs and symptoms, such as a headache.

Acupuncture points were needled bilaterally on body channels except for unilateral needling on channels that bisect along the midline (Ren and Du channels). Needle insertion was at a location and to tissue depth as defined by recognised acupuncture text [21], and the needling sensation known as “*Deqi,*” a sensation experienced by patient and acupuncturist as successful contact between the needle and the correct point [22], was sought on each point, and needles were retained for 20–30 minutes each session. Needles used on all treatments were Vinco brand sterile acupuncture needles in guide tubes (sizes 0.22 × 25 mm, 0.25 × 40 mm, and 0.25 × 75 mm). Additional TCM modalities were included in the treatment and individualised; these included heat applied as appropriate by Teding Diancibo Pu (TDP) Infrared Heat Lamp or smokeless moxibustion. At each session, details of participant signs and symptoms and treatment given were recorded. This included pulse and tongue presentation, changes to basal body temperature (BBT), compliance with exercise and diet, and acupuncture points needled. The acupuncturists included the lead researcher who was TCM trained with 23-year clinical experience and TCM trained practitioners based in Sydney and trained in the study protocols who had a minimum of 3-year clinical experience.

All subjects were free to withdraw from the study at any stage. When participants in either group reported pregnancy, their treatment intervention was terminated. They were asked to confirm pregnancy by blood test.

### 2.4. Outcome Measures

The primary study endpoints were assessed by reported variation in self-knowledge about fertility and ovulation and knowledge of fertile period at 3 months; regularity of menstrual cycle at 3 months displayed on BBT; and reduced menstrual symptoms at 3 months recorded on menstrual record.

Secondary study endpoints included the time from study entry to conception; biochemical pregnancy demonstrated by blood test at six weeks; quality of life changes as measured by MYMOP at three months; and lifestyle change demonstrated by body mass index (BMI) at three months.

Increased awareness of fertility was measured by comparing self-knowledge about fertility and ovulation at intake and exit at three months, and a self-report question of whether their knowledge of when they were fertile had improved and in which way. Menstrual normalisation was documented on a BBT chart issued to participants and they were asked to record their temperature daily prior to rising using the same temperature each day. The chart had an addendum to document menstrual changes, including menses onset and duration, level of pain, menstrual flow, and incidence of clotting, which were also recorded by participating acupuncturists. The incidence of pregnancy was measured by self-report and record of human chorionic gonadotropin (hCG) blood test. The time from study entry to conception was assessed from the date of recruitment to the report of first missed period that resulted in pregnancy. BMI was assessed before and after the intervention.

Measure Your Medical Outcome Profile 2 (MYMOP) [23] was used to measure the quality of life outcomes that the patient considered the most important, including symptoms and desired activities. For the MYMOP2 questionnaire participants are asked prior to intervention to “choose one or two symptoms (physical or mental) which bother you most.” The participants were also asked to “choose one activity (physical, social, or mental) that is important to you and that your problem makes difficult or prevents you doing.”

An adverse event was defined as one that endangers an existing pregnancy, future fertility or the health, and wellbeing of participants. All adverse events (serious and nonserious) were reported to the primary researcher. They were recorded firstly on the treatment record, a copy lodged in a separate adverse event record and analysed and reported.

### 2.5. Sample Size of Pilot Study

Given the lack of research in this area it was difficult to estimate the effect of acupuncture compared to control on changes in lifestyle and pregnancy. Previous research into acupuncture's action in regulating menstrual characteristics suggested a moderate effect size [24]. We expected to detect an absolute improvement in menstrual regularity of 40% from baseline between groups, from 40% in the control group to 80% in the intervention group at the end of the intervention (*p* < 0.05, 80% power). A trial of 56 women was required, 28 women per group, with 80% power at the 5% significance level.

### 2.6. Data Analysis

Quantitative data analysis was undertaken using software program IBM SPSS 20 (IBM SPSS Statistics Version 20). Descriptive analyses including mean, standard deviation, and frequency were undertaken describing the baseline characteristics of participants.

An “intention to treat” analysis was performed. Comparisons were made of primary and secondary outcomes by means of analysis of variance and measures of effect size using relative risks and 95% confidence interval. *p* < 0.05 has been considered statistically significant. An adjusted analysis or covariance was undertaken to assess unexplained or error variance using the variables that were unequal between the two groups, namely, age, duration of fertility problems, and diagnosis of PCOS. For categorical variables a chi-square measure was used and a binary logistic regression undertaken. For continuous variables ANOVA and ANCOVA were used to analyse covariants.

## 3. Results

Over 12 months 160 inquiries were received with 56 women randomised to the study. Of those who met the inclusion criteria and refused to join the study a significant proportion were not willing to undergo randomisation and risk delaying the receipt of an acupuncture intervention for 3 months. As represented in Table 1 the retention rate of participants over the full 3 months in each intervention group was 82% in the acupuncture + lifestyle intervention and 71% in the lifestyle only group. Several potential or actual participants reported moving house or city and it is assumed that at least some of the 21 (37.5% of the total) women lost to follow-up may also have undergone changes, as they were no longer accessible via their work and home contact details. All data collected to the point of exit from the study was analysed.

**Figure 1:**
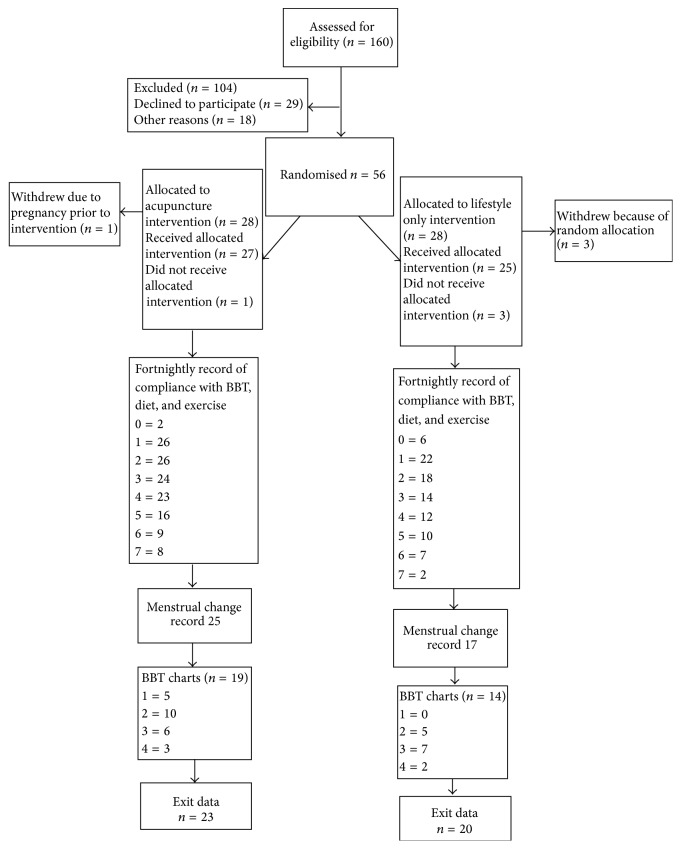
Flowchart of participants through trial.

Baseline characteristics of women are reported in Figure 1. Of the women recruited the average duration of their fertility problems was 4.85 (SD 4.1) years. Twenty-six (46.4%) women had fertility problems solely sourced in female factors; for 24 (42.9%) women their fertility difficulties were unexplained and the balance of six women had combined female-male factors that were being addressed through techniques such as intracytoplasmic sperm injection (ICSI). Nine women were currently enrolled in IVF and further nine reported having tried IVF in the past, 21 had taken clomiphene citrate to stimulate ovulation, and 25 women (44.6%) of the study sample had used no other biomedical treatment prior to the study. Twenty-five women (44.6% of the total group) reported previous use of acupuncture.

Thirty-three women (58.9%) were of English speaking background and the remaining 23 (41.1%) identified themselves as from non-English speaking backgrounds.

The awareness of fertility through identifying ovulation was 28 (50%) indicating that they knew when they ovulated. Of these 13 (46.4%) recognised ovulation by their fertile mucus, seven (25%) by abdominal pain, seven (25%) based on other factors and two (7.2%) by monitoring temperature changes. Twenty-six women (46.4%) monitored their mucus discharge, nine (16.1%) monitored their BBT, and 13 (23.2%) recorded other changes to assist in identifying their fertile period.

In response to the MYMOP questionnaire there was remarkable consistency in the symptoms nominated with a clear dominance of weight loss and tiredness. Surprising, given the purpose of the clinical trial, was the small percentage of these women who nominated infertility and pregnancy failure as their primary concern at that time. Perhaps the women did not perceive fertility as impacting on their wellbeing. It was poignant to read “*I just want to feel good in my body without continuous pain – like I used to be*” on a form which did not encourage lengthy contributions. Several women wrote “depression,” “jealousy,” “anger,” “being emotionally vulnerable,” and “anxiety,” testifying to the difficulty of their lives at that time.

Of the respondents, 37.5% nominated getting more exercise or getting up and moving as their major desired activity.

There was an imbalance between the two study groups for two variables at baseline: the duration of infertility and biomedical diagnosis; these were adjusted for in the analysis.

## 4. Primary Study Endpoints

As indicated in Table 2 there was a statistically significant increase in fertility awareness in the group of women who received acupuncture (86.4%, 19) compared to 40% (*n* = 8) of the lifestyle only participants, (Relative Risk (RR) 2.38, 95% confidence interval (CI) of 1.25, 4.50), with an adjusted *p* value of 0.011 (Table 5).

There was no significant change in menstrual regularity over time between the two groups; however, the participants receiving acupuncture trended toward retaining regularity over 3 cycles (of 71.4%) whereas the lifestyle only group lost regularity (71.4% to 50%). The low numbers of responses of 3rd-cycle data limit the value of interpreting this trend. There were no statistically significant differences in the length of the follicular and ovulatory phases of the menstrual cycle over three months between groups.

## 5. Reduced Menstrual Symptoms

There were no statistically significant differences between groups in other menstrual characteristics including length of cycle [Table 3], presence of menstrual clots, and menstrual pain.

There was no statistical difference in the pregnancy rate between groups with seven women (adjusted *p* = 0.992) achieving a pregnancy during the course of the study intervention. Those receiving the acupuncture conceived within an average of 5.5 weeks compared to 10.67 weeks for the lifestyle only group (*p* = 0.422). For those who received acupuncture this is effectively half the time to conception. There is a trend in the follow-up data indicating a difference in the numbers of pregnancies in the 12 months following trial completion: 10 women in total in the acupuncture group became pregnant and 5 in the lifestyle only group, with the number of live births also varying to 80% in the acupuncture group and 60% in the lifestyle only group (*p* = 0.176).

Quality of life improved for women receiving acupuncture [Table 4]. In relation to changes in desired activity the acupuncture intervention participants recorded a change of 1.80 (1.2) compared to the lifestyle only group of 0.94 (1.2). This represented a statistically significant adjusted *p* value of 0.047. The MYMOP measure of changes in wellbeing also showed significant differences between the two groups: 0.95 (1.4) in the acupuncture group and 0.05 (1.4) in the lifestyle only group. This represented a statistically significant adjusted *p* value of 0.042.

There was no significant difference in BMI in conclusion of the intervention for women in the lifestyle only group compared with the acupuncture group.

Analysis of attendance for acupuncture shows that only 67% received the full course of 9 treatments with a mean of 7.62 and standard deviation of 2.5. Four trial participants randomised to receive acupuncture completed either part or all treatments and then failed to agree to an exit interview. Of the 28 women one was excluded because she fell pregnant prior to receiving any acupuncture; 3 withdrew after receiving less than the prescribed 9 acupuncture treatments (2 of these were lost to follow-up because they moved outside the area); 2 women completed their acupuncture (administered by acupuncturists other than the researcher) but would not respond to requests for an exit interview and they also became lost to follow-up. This represents 6 women (21%) either not completing the intervention or leaving the trial reporting requirements incomplete.

Two women randomised to the lifestyle only intervention telephoned within a few days of recruitment to withdraw from the study. In all, 8 women (28.6%) were lost to follow-up in the lifestyle only group although some partial data from some of these women were able to be included in the analysis. Of the women who completed the acupuncture intervention none expressed dissatisfaction with the intervention despite some not complying with data collection measures.

The average number of days in treatment was 64.74 (SD 24.1). In actuality only 4 (14.3%) women received treatment over 84 days or more (being equivalent to 3 menstrual cycles assuming an average length of menstrual cycle of 28 days).

Eighteen women (nearly 82 percent) reported no side effects during the acupuncture intervention. Three women (13.6%) reported side effects which were nominated as “*slight bruising on some points,*” “*headaches in the first three sessions which decreased in severity, felt very emotional (more than usual),*” “*awareness of old buried feelings,*” and “*a little pain and occasional bruising,*” There were no reported adverse events from the lifestyle only participants.

## 6. Discussion

The main findings were that this acupuncture intervention, compared to lifestyle only, resulted in significant increases in fertility awareness and quality of life measures in relation to wellbeing; it increased the ability of the recipients to engage in desired activities, such as exercise or rest, and it shortened the time to conception by half. The findings provide preliminary evidence that the acupuncture intervention is acceptable and is not inert and that acupuncture dose may have a significant influence on outcomes. It was also apparent that the lifestyle only intervention was less acceptable to this population and as a comparator to acupuncture. The measurement tools to identify physiological changes, particularly menstrual change, require greater refinement to make adherence easier for the participant and to improve the researcher's capacity to analyse the data.

Estimating the treatment effect in this pilot study was difficult due to small numbers in the sample and the study being underpowered. Calculated against the measure that meant most to the participants, pregnancy, the power of the study was 3%. Analysing poststudy pregnancy rates the achieved power was 33% (calculated on the G^*∗*^Power program). Several effects were evident in the study, specifically in awareness, wellbeing, capacity to undertake desired activity, and time to conception. Changes in menstrual indicators were not significant; however, this may have resulted from both poor record keeping and reporting by participants and an inadequate dose of acupuncture that did not cover at least 3 menstrual cycles. This also may have been influenced by the high proportion of women with PCOS.

A more fully powered study is justified on the basis of results, including trends and indications, displayed by this underpowered feasibility study. On the measure of pregnancy a fully powered study would require a sample size of 189 (calculated on the dropout rate of 12.5% in this study added to 168 participants to achieve full power) (G^*∗*^Power). The clear benefits to the women involved of increased awareness of their fertility, their improved sense of wellbeing, and being able to engage in desired activities may flow on to improved fertility outcomes. There are trends that indicate this may be the case: reduced time to conception, reduced incidence of menstrual clotting, reduced length of pain and intensity of pain, normalisation of the length of the menstrual cycle, and the increased heaviness of menstrual flow in the acupuncture intervention participants. Cai et al. [25] that undertook a prospective analysis of cases using a standard acupuncture protocol with a nonacupuncture control concluded that although “acupuncture did not increase the cumulative pregnancy rate, it decreased the time to achieve pregnancy” in subsequent IVF cycles. Although indications are that diet and exercise regimes may have positive effects on female fertility, studies where acupuncture and such lifestyle changes are used as comparators were not located by the authors.

The strengths of the study were that it addressed an underexplored research area using acupuncture to influence women's reproductive cycles outside ART; it used an intervention that approximated usual clinical practice of acupuncture; the participants welcomed the intervention and reported few adverse events. The limitations were that the study was underpowered and a sample size of 189 would be required to use pregnancy as an outcome measure. The adequacy of lifestyle modification as the comparator has not been established in other studies and this study indicated that measures to retain participants in the control group and ensure their commitment to fully complete assessments were inadequate. Because of the nature of an acupuncture pragmatic trial, participants were not blind to allocation.

## 7. Conclusion

The multiphasic fertility acupuncture protocol tested in this trial did positively influence the women who received it compared to the women who used lifestyle modification alone. It increased their fertility awareness and improved their wellbeing. Those who conceived did so in half the time of their lifestyle only peers. As a pilot study it offers a framework for greater exploration of acupuncture's capacity to influence women's reproduction but would require modification to include a more credible and acceptable control for this study population.

## Supplementary Material

The acupuncturists who delivered the trial intervention received instructions as documented in the Supplementary Materials.

## Figures and Tables

**Table 1:** Extract of guidelines issued to trial acupuncturists.

Frequency	Weekly			

Number of treatments	9			

Timing	1 hour [include front (anterior) and back (posterior) treatment per session]			

Point location	As per Deadman's *A Manual of Acupuncture*			

Needle depth	As per Deadman's *A Manual of Acupuncture*			

Manipulation	Achieve deqi on insertion and renew qi sensation 10–15 minutes after insertion			

Retention time	20–30 minutes			

Needling	Bilateral unless on Ren and Du channels			

Needles	Use needles supplied by Helio Supply Co., that is, AcuGlide and Vinco			

Heat	If heat is necessary, apply using TDP lamp or smokeless moxa			

Differential diagnosis relating to phase of menstrual cycle	Phase 1 during period	Phase 2 after period	Phase 3 during ovulation	Phase 4 after ovulation

Core points [14]	Sp 10, 6, 8 LI 4 St 28Ki 14	Ren 4, 7 Ki 3, 4, 5, 6, 8, 13 St 27, 30, 36 Bl 23, 32 Liv 3 Sp 4, 6, 10	Liv 3, 5 Ki 13, 14, 8, 5, 4 Sp 13, 8, 6, 5 Pc 6, 5 Ht 7, 5 Yintang Zigong GB 26	(A) [boost yang by supplementing yin] Ren 2, 4, 5, 7, 15 Ki 3, 6 Bl 23 (B) [boost yang promoting qi] Ren 4, 5, 6, 12 St 25, 36 Sp 6 Ki 3 Bl 20, 23 (C) [boost yang by nourishing blood] Ren 4, 12 St 36 Sp 6, 10 Ki 5 Bl 17

Examples of other commonly used acupoints	*Shen* (emotional) disorders	GV 20, Yintang, Ht 7, Pc 6, Kidney chest pts: Ki 23, 24, 25		
	Ovulation failure	St 29, Ren 4, 3 Zigong, LI 4, Sp 6		
	Biomedical diagnosis, PCOS	Sp 6, Zigong, Ren 3, 4, Bl 20, 23, 18		

Termination of treatment	On pregnancy			
	For 2 weeks after embryo transfer or on negative pregnancy test			

**Table 2:** Baseline characteristics of women at trial entry to allocated treatment group.

	Acupuncture intervention *n* = 28	Lifestyle only intervention *n* = 28	*p* value
Age (years)	33.14 (5.4)	33.93 (5.0)	0.572
Fertility history			
Gravida = 0 *n* (%)	11 (39.3)	7 (25)	0.252
Parity = 1 or >1 *n* (%)	8 (28.6)	7 (32.1)	0.771
Duration of infertility			
Mean (SD)	3.9 (3.4)	5.8 (4.6)	0.080^*∗*^
Reasons for infertility			
Female factor *n* (%)	15 (53.6)	11 (39.3)	
Unexplained	10 (35.7)	13 (26.4)	
Unknown, combined + male factor	3 (10.7)	4 (14.3)	
Biomedical fertility diagnosis			
PCOS *n* (%)	12 (42.9)	9 (32.1)	0.408
Unknown *n* (%)	10 (35.7)	14 (50)	
Other *n* (%)	6 (21.4)	5 (17.9)	
BMI			
Combined mean (SD)	30.0 (9.8)	30.3 (7.4)	0.893
Previous use of acupuncture *n* (%)	13 (46.4)	12 (42.9)	0.788
Demographic details			
Employment *n* (%)			
Working full time	17 (60.7)	16 (57.1)	0.528
Working part time	8 (28.6)	6 (21.4)	
Unemployed/home duties	2 (7.1)	6 (17.9)	
Education status *n* (%)			
School incompletion	0	2 (7.1)	
School/TAFE completion	16 (57.1)	13 (46.4)	0.309
Tertiary completion	12 (42.9)	13 (46.4)	
Racial origin *n* (%)			
Caucasian	16 (57.1)	19 (67.9)	0.408
English speaking background (ESB)	15	18	0.415
NESB	13	10	0.275
Marital status *n* (%)			
Married	25 (89.3)	22 (78.6)	
De facto	3 (10.7)	6 (21.4)	

*∗* indicates possible significance, for example, *p* < 0.05.

**Table 3:** Primary study endpoints by treatment group.

	Acupuncture intervention *N* = 28	Lifestyle only intervention *N* = 28	*p* value	Adjusted analysis	RR/CI (CI 95%)
Increase in fertility awareness (%)	19 (86.4)	8 (40)	0.002^*∗*^	0.011^*∗*^	2.38 (1.25, 4.50)
Regularity of cycle *n* (%)					
At entry (%) *n* = 56	20 (71.4)	20 (71.4)			
M1 *n* = 43	16 (61.5)	12 (70.6)	0.543	0.823	0.87 (0.57, 1.34)
M2 *n* = 31	12 (60)	5 (45.5)	0.436	0.262	1.32 (0.63, 2.77)
M3 *n* = 15	5 (71.4)	4 (50)	0.398	0.252	1.43 (0.65, 3.30)
Menstrual details (mean/SD)					
Length of period					
M entry *n* = 56	4.86 (2.3)	4.85 (1.5)	0.984		
M1 *n* = 36	5.39 (2.5)	5.08 (1.1)	0.671	0.383	0.31 (−1.18, 1.8)
M2 *n* = 30	5.11 (2.0)	4.33 (1.4)	0.255	0.631	0.78 (−0.77, 2.33)
M3 *n* = 18	5.11 (1.3)	4.67 (1.3)	0.478	0.452	0.44 (−0.85, 1.73)
M exit *n* = 39	4.52 (2.2)	4.72 (2.2)	0.783	0.764	−0.2 (−1.65, 1.25)
Length of cycle					
M entry *n* = 56	34.64 (18.3)	35.43 (18.4)	0.873		
M1 *n* = 34	32.52 (13.9)	31.00 (6.2)	0.713	0.360	1.52 (−6.8, 9.84)
M2 *n* = 29	29.12 (4.0)	29.25 (8.9)	0.957	0.802	−0.13 (−5.15, 4.89)
M3 *n* = 16	29.43 (2.6)	26.78 (3.1)	0.091	0.996	2.65 (−0.48, 5.78)
M exit *n* = 37	29.05 (11.9)	30.65 (6.4)	0.624	0.285	−1.6 (−8.15, 4.95)
Nature of menstrual flow (%)					
Heavy			*Chi-square* M entry 0.716 M1 0.077 M2 0.009^*∗*^ M3 0.819		
M entry *n* = 56	11 (39.3)	12 (42.9)			0.92 (0.49, 1.72)
M1 *n* = 41	11 (44)	2 (12.5)			3.52 (0.89, 13.85)
M2 *n* = 29	11 (55)	1 (11.1)			4.95 (0.75, 32.76)
M3 *n* = 14	2 (28.6)	2 (28.6)			1 (0.19, 5.25)
Moderate					
M entry *n* = 56	9 (32.1)	11 (39.3)			0.82 (0.4, 1.66)
M1 *n* = 41	10 (40)	8 (50)			0.8 (0.4, 1.59)
M2 *n* = 29	6 (30)	3 (33.3)			0.9 (0.29, 2.82)
M3 *n* = 14	3 (42.9)	2 (28.6)			1.5 (0.35, 6.4)
Light					
M entry *n* = 56	5 (17.9)	4 (14.3)			1.25 (0.37, 4.17)
M1 *n* = 41	4 (16)	6 (37.5)			0.43 (0.14, 1.28)
M2 *n* = 29	1 (5)	5 (55.6)			0.09 (0.01, 0.66)
M3 *n* = 14	2 (28.6)	3 (42.9)			0.67 (0.16, 2.84)
Inconsistent					
M entry *n* = 56	3 (10.7)	1 (3.6)			3 (0.33, 27.12)
M1 *n* = 41	0	0			
M2 *n* = 29	2 (10)	0			
M3 *n* = 14	0	0			

*∗* indicates possible statistical significance, for example, *p* < 0.05.

**Table 4:** Primary study endpoints by treatment group (C).

	Acupuncture intervention *N* = 28	Lifestyle only intervention *N* = 28	*p* value	Adjusted analysis	RR/CI (CI 95%)
Incidence of menstrual clots (%)					
None					
M entry *n* = 56	10 (35.7)	9 (32.1)	M entry 0.556		1.11 (0.53, 2.31)
M1 *n* = 39	11 (45.8)	11 (73.3)	M1 0.221		0.63 (0.37, 1.06)
M2 *n* = 28	9 (47.4)	7 (77.8)	M2 0.287		0.61 (0.34, 1.1)
M3 *n* = 13	6 (85.7)	4 (66.7)	M3 0.188		2.14 (0.95, 4.85)
Small					
M entry *n* = 56	12 (42.9)	9 (32.1)			1.33 (0.67, 2.65)
M1 *n* = 39	8 (33.3)	3 (20)			1.67 (0.52, 5.31)
M2 *n* = 28	7 (36.8)	1 (11.1)			3.32 (0.48, 23.06)
M3 *n* = 13	1 (14.3)	0			
Large					
M entry *n* = 56	6 (21.4)	9 (32.1)			0.67 (0.27, 1.62)
M1 *n* = 39	5 (20.8)	1 (6.7)			3.13 (0.4, 24.22)
M2 *n* = 28	3 (15.8)	1 (11.1)			1.42 (0.17, 11.83)
M3 *n* = 13	0	2 (33.3)			
Variable					
M entry *n* = 56	0	1 (3.6)			
Incidence of menstrual pain					
M entry *n* = 56	23 (82.1)	20 (71.4)	0.342		1.15 (0.86, 1.54)
M1 *n* = 40	17 (68)	5 (33.3)	0.033^*∗*^	0.056	2.04 (0.95, 4.38)
M2 *n* = 30	10 (50)	3 (30)	0.297	0.430	1.67 (0.59, 4.73)
M3 *n* = 13	2 (28.6)	3 (50)	0.529		0.67(0.17, 2.67)
Length of menstrual pain (mean (SD))					
M entry *n* = 56	1.7 (1.6)	1.2 (1.0)	0.669		
M1 *n* = 40	1.46 (1.7)	0.63 (1.4)	0.108	0.238	0.83 (−0.2, 1.86)
M2 *n* = 36	0.9 (1.2)	0.47 (1.1)	0.271	0.258	0.43 (−0.36, 1.22)
M3 *n* = 15	0.57 (0.5)	0.63 (1.1)	0.906	0.587	−0.06 (−1.02, 0.9)
Intensity of menstrual pain (mean (SD))					
M entry *n* = 56	3 (1.3)	2 (1.4)	0.261		
M1 *n* = 23	3 (1.3)	2 (1.4)	0.146	0.285	1 (−0.37, 2.37)
M2 *n* = 14	1.91 (1.4)	2.67 (1.5)	0.423	0.141	−0.76 (−2.76, 1.24)
M3 *n* = 7	1.5 (1.0)	2.67 (1.5)	0.272	0.840	−1.17 (−3.6, 1.26)

*∗* indicates possible statistical significance, for example, *p* < 0.05.

**Table 5:** Secondary study endpoints by treatment group.

	Acupuncture intervention *N* = 28	Lifestyle only intervention *N* = 28	*p* value	Adjusted analysis
Pregnancy (%)				
Incidence *n* = 43	4 (16)	3^1^ (15)	0.927	0.992
Time from study entry to conception (weeks) *n* = 43	5.5	10.67	0.422	0.452
Quality of life changes (MYMOP) *n* = 43 (mean (SD))				
Changes in symptom 1	1.27 (2.1)	1.55 (1.5)	0.628	0.775
Changes in symptom 2	1.81 (1.4)	1.40 (1.1)	0.305	0.291
Changes in activity	1.80 (1.2)	0.94 (1.2)	0.033^*∗*^	0.047^*∗*^
Changes in wellbeing	0.95 (1.4)	0.05 (1.4)	0.043^*∗*^	0.042^*∗*^
Change in MYMOP profile score	1.47 (1.1)	1.03 (0.8)	0.156	0.165
Lifestyle change (BMI)				
Change in BMI (mean (SD))	0.0318 (1.1)	−0.75 (1.3)	0.475	0.585
Sustained lifestyle change at 12 months *n* = 31 (%)	10 (53)	6 (50)	0.886	
Acceptability of intervention *n* (%)				
Completion of exit interview	22 (78.57)	20 (71.43)	0.314	
Record of menstrual changes	26 (92.86)	16 (57.14)	0.677	

^1^One of these pregnancies was achieved through IVF due to male factor problems for the couple. The participant advised the researcher after her pregnancy that she had used acupuncture prior to this conception, her first ever after years of IVF cycles.

*∗* indicates possible statistical significance, for example, *p* < 0.05.
