# Corrigendum to “Prior to Conception: The Role of an Acupuncture Protocol in Improving Women's Reproductive Functioning Assessed by a Pilot Pragmatic Randomised Controlled Trial”

**DOI:** 10.1155/2018/2343604

**Published:** 2018-05-02

**Authors:** Suzanne Cochrane, Caroline A. Smith, Alphia Possamai-Inesedy, Alan Bensoussan

**Affiliations:** ^1^School of Science & Health, Western Sydney University, Locked Bag 1797, Penrith, NSW 2751, Australia; ^2^National Institute of Complementary Medicine, Western Sydney University, Locked Bag 1797, Penrith, NSW 2751, Australia; ^3^School of Social Science & Psychology, Western Sydney University, Locked Bag 1797, Penrith, NSW 2751, Australia

In the article titled “Prior to Conception: The Role of an Acupuncture Protocol in Improving Women's Reproductive Functioning Assessed by a Pilot Pragmatic Randomised Controlled Trial” [1], the Conflicts of Interest section should read as follows:

CompleMED, a precursor of the National Institute of Complementary Medicine, where Professor Caroline A. Smith and Professor Alan Bensoussan are based as researchers, received a donation in 2010 of $20,000 from the Acupuncture IVF Support Pty Ltd. This donation was untied and applied towards other unrelated research.
