# Supplementary material for: Prior to Conception: The Role of an Acupuncture Protocol in Improving Women's Reproductive Functioning Assessed by a Pilot Pragmatic Randomised Controlled Trial
Source: Evid Based Complement Alternat Med. 2016 May 3;2016:3587569. doi: 10.1155/2016/3587569 (PMC4868913; doi:10.1155/2016/3587569)
Supplement: Supplementary file 1 — The acupuncturists who delivered the trial intervention received instructions as documented in the Supplementary Materials. [file 3587569.f1.docx]

**Acupuncture Treatment Protocols**

| Frequency | Weekly |
| --- | --- |
| Number of treatments | 9 |
| Timing | 1 hour [include front(anterior) & back(posterior) treatment per session] |
| Point location | as per Deadman’s **A Manual of Acupuncture** |
| Needle depth | as per Deadman’s **A Manual of Acupuncture** |
| Manipulation | Achieve deqi on insertion, renew qi sensation 10-15 minutes after insertion |
| Retention time | 20-30 minutes |
| Needling | Bilateral unless on Ren & Du channels |
| Needles | Use needles supplied by Helio Supply Co., that is, Acuglide & Vinco |
| Heat | If heat is necessary apply using TDP lamp or smokeless moxa |
| Treatment principles | 1. by TCM diagnosis 2. according to time of menstrual cycle 3. according to emotional state or shen presentation 4. biomedical condition 5. add points according to presenting signs & symptoms |
| Termination  of treatment | On pregnancy  For 2 weeks after embryo transfer or on negative pregnancy test |

Select an appropriate set of acupuncture points according to **time in cycle** and **TCM diagnosis** from the table below:

Treatment according to time of cycle should make special note of the beginning and end of the cycle. Giving acupuncture during the period and just prior to the period should only be undertaken if considered appropriate; that is, if there is period pain or clotting then acupuncture during the period is necessary or if there is a likelihood of pregnancy cautious treatment only should be used prior to the period.

| Diff Dx/ Phase | Phase 1  During period | Phase 2  Post-period | Phase 3  Ovulation | Phase 4  Post-ovulation |
| --- | --- | --- | --- | --- |
| Core points(Lyttleton 2004) | Sp 10, 6,8  LI 4  St 28  Ki 14 | Ren 4,7  Ki 3,4,5,6,8,13  St 27,30,36  Bl 23,32  Liv 3  Sp4,6,10 | Liv 3,5  Ki 13,14,8,5,4  Sp 13,8,6,5  Pc 6,5  Ht 7,5  Yintang  Zigong  GB 26 | **A.** [boost yang by supplementing yin]  Ren 2,4,5,7,15  Ki 3,6  Bl 23  **B.** [boost yang promoting qi]  Ren 4,5,6,12  St 25,36  Sp 6  Ki 3  Bl 20, 23  **C.** [boost yang by nourishing blood]  Ren 4,12  St 36  Sp 6,10  Ki 5  Bl 17 |
| Kidney yin xu |  | + Ki 2 | + Ren 4  Ki 3,6 | A. + Ki 2 |
| Kidney yang xu |  | + Bl 23  Ren 3  Ren 2  Bl 32 | + Ren 6,4  St 29 | + moxa |
| Kidney jing xu |  | + Ki 12 | + Ren 4  Ki 12  St 27 | A pts |
| Liver / Heart Stagnation |  | + Liv 2  Ht 5,7  Pc 6,7 | + | C pts  + Liv 2,3,4,5,8,9,11  Pc 5,6,7  Ren 3 |
| Blood stagnation |  | + Ki 16  Ren 3  Sp 12  St 29  Liv 5  Sp 8 | + St 29  Sp 10  Liv 8  Bl 17 | C pts  + St 28,29  Liv 8,5  Sp 6,8  Ki 4,5  baliao |
| Phlegm-Damp |  | + GB 26  Sp 5  Bl 28 | + Bl 22,28  GB 27,28  Sp 9  Ki 7  St 29 | B pts  + Ren 3, 6,9  GB 27,28  Bl 32  Sp 6,9 |
| Shen disorder | + GV 20, Yintang, Ht 7, Pc 6, Kidney chest pts: Ki 23, 24, 25  Pts related to each element, eg.: Wood (GB 13,24,40, Liv 2,13,14); Fire (Ht 4,7, SI 11,17 Pc 1,2, SJ 10,23); Earth (St 8,25,40 Sp 4,15) Metal (Lu 1,2,3 LI 17,18) Water (Bl 10,52, Ki 1,21,23,24,25)  Ren, Du mai & extra pts: Ren 1,4, 6,17,22, Du 4,10,20,24, yintang(Hicks, Hicks et al. 2004) | + GV 20, Yintang, Ht 7, Pc 6, Kidney chest pts: Ki 23, 24, 25  Pts related to each element, eg.: Wood (GB 13,24,40, Liv 2,13,14); Fire (Ht 4,7, SI 11,17 Pc 1,2, SJ 10,23); Earth (St 8,25,40 Sp 4,15) Metal (Lu 1,2,3 LI 17,18) Water (Bl 10,52, Ki 1,21,23,24,25)  Ren, Du mai & extra pts: Ren 1,4, 6,17,22, Du 4,10,20,24, yintang(Hicks, Hicks et al. 2004) | + GV 20, Yintang, Ht 7, Pc 6, Kidney chest pts: Ki 23, 24, 25  Pts related to each element, eg.: Wood (GB 13,24,40, Liv 2,13,14); Fire (Ht 4,7, SI 11,17 Pc 1,2, SJ 10,23); Earth (St 8,25,40 Sp 4,15) Metal (Lu 1,2,3 LI 17,18) Water (Bl 10,52, Ki 1,21,23,24,25)  Ren, Du mai & extra pts: Ren 1,4, 6,17,22, Du 4,10,20,24, yintang(Hicks, Hicks et al. 2004) | + GV 20, Yintang, Ht 7, Pc 6, Kidney chest pts: Ki 23, 24, 25  Pts related to each element, eg.: Wood (GB 13,24,40, Liv 2,13,14); Fire (Ht 4,7, SI 11,17 Pc 1,2, SJ 10,23); Earth (St 8,25,40 Sp 4,15) Metal (Lu 1,2,3 LI 17,18) Water (Bl 10,52, Ki 1,21,23,24,25)  Ren, Du mai & extra pts: Ren 1,4, 6,17,22, Du 4,10,20,24, yintang(Hicks, Hicks et al. 2004) |
| Extraordinary meridian presentation | Opening & closing pts as appropriate, eg. Chongmai Sp 4 + Pc 6 |  |  |  |

In the event of a specific **biomedical diagnosis** consider including the following points:

Acupuncture for use with specific biomedical diagnoses

| Condition |  |
| --- | --- |
| Tubal infertility | Zigong, BL 32, ST 30, ST 29, SP 12, KI 12. (Wang and Li 2005) |
| Endometriosis | **Post-period**:  Ki 13, 14, 18 Ren 3,4,7,12 Sp 6 Liv 8  **Ovulation**:  Liv 3, 11 Sp 6,4,12,13 St 29 Ki 4,8 Pc 6  Ht 7 Zigong  **Post-ovulation:**  Ren 4 Ki 3 St 29 Bl 23 Pc 7 Liv 2 Sp 1 Ht 7  **Period:**  St 28,29 Sp 12,13,8,6,10 Ki 14 Ren 6  Bl 31-34, 26, 28,22 Shiqizhuixia Liv 2,8 Li 4 Pc 5(Lyttleton 2004)  Bl 18,20,23, Liv 13,14, GB 25(Chen, Yue et al. 1996) |
| Ovulation failure | St 29, Ren 4,3 Zigong, LI 4, Sp 6  . Ki xu + Bl 23, Ki 3  .liver constraint + Liv 3  .blood xu + Sp 10  .phlegm-damp + Sp 9, St 40  .blood stasis + Bl 17(Chen and Li 2008) |
| Anovulation or delayed ovulation | Ren 3, Sp 10 & Ki 12(Zheng and Qian 2002) |
| Salpingemphraxis/  blocked tubes | Sp 6, Zigong, Ren 6, 3, LI 4, Ki 3  .qi stagnation & blood stasis + Bl 17,18 Liv 3  .damp-heat & stasis Ren 3 (xiefa)  .cold-damp & stasis + moxa to Bl 21  .phlegm & stasis + St 40 (ping bu ping xiefa)(Chen and Li 2008) |
| PCOS | Sp 6, Zigong, Ren 3,4, Bl 20,23,18  .if obese use Ren 4,6, 10, 12 Sp 15, St 24, St 26 |
| Immune infertility | Bl 15,17,18,23, Liv 3, Sp 10, Ki 3, Ht 7 (ping bu ping xie)(Chen and Li 2008) |
| Premature ovarian failure | Ren 3,4,6,10,12 zigong, Ki 12, Bl 23, jiaji T5-L4  .liver & kidney xu + Sp 6,9, Bl 18, Ht 6, Ki 7  .spleen & kidney yang xu + Bl 20,32, Du 4, Sp 8(Chen and Li 2008) |

**Guidelines for choosing acupuncture points for treatments**

A TCM diagnosis will be provided following subject’s intake interview with Sue Cochrane. Do not change this diagnosis without consultation with Sue first.

1^st^ choose points suited to the time of the menstrual cycle

Eg. Zigong for ovulation time or

Sp10 during period

2^nd^ choose points suited to the TCM diagnosis

Eg. Ren 4 + Ki 3 for Kidney yin xu

3^rd^ choose points suited to the biomedical diagnosis

Eg. Sp 6 for PCOS

4^th^ choose points on presenting symptoms

Eg. GB 20 for occipital headache
